# Self-Consistent Framework Connecting Experimental Proxies of Protein Dynamics with Configurational Entropy

**DOI:** 10.1021/acs.jctc.8b00100

**Published:** 2018-05-25

**Authors:** Markus Fleck, Anton A. Polyansky, Bojan Zagrovic

**Affiliations:** Department of Structural and Computational Biology, Max F. Perutz Laboratories, University of Vienna, Campus Vienna Biocenter 5, Vienna 1030, Austria

## Abstract

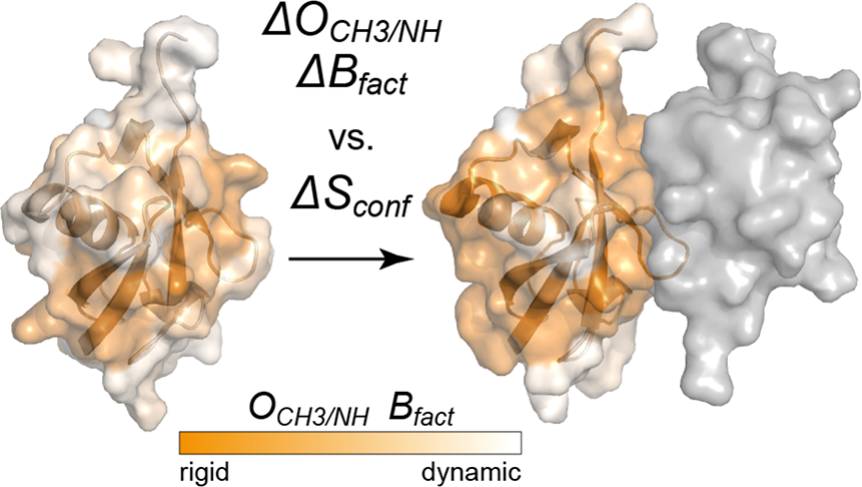

The recently developed NMR techniques enable estimation of protein configurational entropy change from the change in the average methyl order parameters. This experimental observable, however, does not directly measure the contribution of intramolecular couplings, protein main-chain motions, or angular dynamics. Here, we carry out a self-consistent computational analysis of the impact of these missing contributions on an extensive set of molecular dynamics simulations of different proteins undergoing binding. Specifically, we compare the configurational entropy change in protein complex formation as obtained by the maximum information spanning tree approximation (MIST), which treats the above entropy contributions directly, and the change in the average NMR methyl and NH order parameters. Our parallel implementation of MIST allows us to treat hard angular degrees of freedom as well as couplings up to full pairwise order explicitly, while still involving a high degree of sampling and tackling molecules of biologically relevant sizes. First, we demonstrate a remarkably strong linear relationship between the total configurational entropy change and the average change in both methyl and backbone-NH order parameters. Second, in contrast to canonical assumptions, we show that the main-chain and angular terms contribute significantly to the overall configurational entropy change and also scale linearly with it. Consequently, linear models starting from the average methyl order parameters are able to capture the contribution of main-chain and angular terms well. After applying the quantum-mechanical harmonic oscillator entropy formalism, we establish a similarly strong linear relationship for X-ray crystallographic B-factors. Finally, we demonstrate that the observed linear relationships remain robust against drastic undersampling and argue that they reflect an intrinsic property of compact proteins. Despite their remarkable strength, however, the above linear relationships yield estimates of configurational entropy change whose accuracy appears to be sufficient for qualitative applications only.

## 1 Introduction

Noncovalent interactions involving biomolecules are the basis of a large number of fundamental biological processes including transcription, translation, cell signaling, and many others.^1^ In an isothermal–isobaric ensemble with a constant number of particles (NPT), the Gibbs free energy change
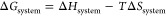
1determines the probability of such an interaction to occur and is thus of central importance. While the enthalpic (Δ*H*_system_) contributions to the free energy change upon binding are well understood and are frequently exploited in contexts such as computational drug design,^2−5^ the entropic component of the free energy change remains in the background. However, a number of different fields ranging from bioengineering to rational drug design would benefit strongly from a comprehensive understanding of entropic contributions to biomolecular interactions.^3−5^ Historically, the dominant entropic term in biomolecular interactions has been attributed to solvent entropy change,^6^ accounting for the hydrophobic effect. However, recent experimental evidence,^7−10^ using NMR-derived methyl order parameters^11^ to probe protein dynamics, suggests that the change in protein configurational entropy can be of comparable magnitude to solvent entropy change and, thus, drastically influence the thermodynamics of binding. While these pioneering studies have opened up experimental access to protein configurational entropy change, they are nevertheless fraught with several underexplored difficulties. First, such approaches do not measure contributions from coupled, correlated dynamics in proteins directly, but reconstruct them from empirical, linear fits across sets of reference proteins. While the necessary linearity of the coupling corrections with the total configurational entropy change has been demonstrated computationally in the case of torsional side-chain rotamer degrees of freedom,^12^ its validity for other contributions is still not clear. Second, the central assumption behind the above approaches, i.e., that of a linear dependence between the average NMR order parameter change and the configurational entropy change, has been shown for several model potentials (e.g., refs (13−16)), including the harmonic and square-well potentials. However, a direct analysis from actual simulations of the relationship between the change in the average order parameters and the total configurational entropy change, including all contributions, has never before been carried out. This, in particular, concerns the contribution of the potentially significant main-chain and angular dynamics, although previous work has suggested that the two contributions may be negligible.^10,12,17^ Moreover, it is not clear how the experimental configurational entropy change estimation is affected by the fact that in the experiment only a limited number of methyl order parameters can be measured, while, on the other hand, they need to report on the collective behavior of many more degrees of freedom. Finally, methyl order parameters from soft side-chain torsions have been demonstrated to be insensitive to the broadness of torsional energy wells.^12^ While there is evidence that the associated vibrational entropy could be more significant than originally proposed^18^ in the case of side-chain burying^19^ and drug-like ligand binding,^20^ a direct examination of its overall impact in the case of a diverse set of protein complexes is still missing.

Following these considerations, one would like to formulate a strategy to simultaneously assess the impact of the above-mentioned terms, analyze the quality of the linear relationships involved, and potentially also identify novel experimental proxies of configurational entropy. A promising approach in this regard entails a self-consistent comparison between the changes in different experimental observables and the total configurational entropy change and its components, all derived from the same protein ensembles generated using molecular dynamics (MD) simulations. An analogous approach, employing MD ensembles to link the dynamical attributes of molecules with experimental observables, was recently used in order to quantify positional uncertainties, rather than configurational entropy, in structures of small molecules derived from NMR-crystallographic chemical shifts.^24^

In general, computational approaches for calculating configurational entropy^25−35^ are capable of accounting for couplings up to pairwise order. Furthermore, they are independent of any empirical, *a priori* assumptions concerning the relationship between the sampled parameters and configurational entropy and can treat all contributions in the system explicitly. Also, as configurational entropy is directly calculated for the macromolecules alone, there is no need to estimate the change in solvent entropy. While such approaches may suffer from force field-related issues and incomplete sampling, a self-consistent analysis of entropies and entropy proxies on the same ensemble is likely to be highly informative in any case.

A powerful approach for deriving configurational entropy from simulated ensembles is the maximum information spanning tree (MIST) approximation,^29,36^ a variant of the mutual information expansion (MIE) method,^30,31,33^ which unifies several advantages. First, by directly sampling probability densities, MIST (as MIE) includes the contribution of anharmonicities and nonlinear couplings up to second order without any additional assumptions. Second, unlike MIE, MIST yields a mathematically guaranteed upper bound to configurational entropy and is less likely to overestimate the contribution from pairwise couplings, which is directly related to its better convergence behavior. Note, however, that for small molecules, spurious coupling, especially relevant for the MIE approach, can be suppressed,^37^ and, in this way, the MIE approach can converge more readily to the final result than MIST.^38^ Furthermore, the direct sampling of probability densities up to arbitrary spatial resolution, paired with high temporal resolution of MD simulations, leads to a natural treatment of vibrational entropy by MIST (as well as MIE). Last but not least, separate contributions to the configurational entropy can be extracted from the analysis, mostly without significant effort.

Our recent development of a parallel program suite for the calculation of configurational entropy^39^ using both MIST and MIE formalisms, canonically applied in bond–angle–torsion (BAT) coordinates,^31,40−45^ has now for the first time created an opportunity for a self-consistent investigation of the relationship between NMR methyl order parameter change and the total configurational entropy change on a large set of simulated protein complexes combined with exhaustive sampling. The same approach can also be used to study the equivalent relationship for other standard NMR proxies of protein dynamics, e.g., backbone-NH order parameters. Indeed, NH order parameters have been employed to assay configurational entropy,^46,47^ but their application is less common in this context. This is arguably due to the fact that they are experimentally more difficult to capture as compared to methyl order parameters. Importantly, there still does not exist a consensus on the overall contribution of main-chain dynamics on the configurational entropy change upon protein binding. For example, a recent computational study of the bovine pancreatic trypsin inhibitor protein has shown that this contribution may be significant.^38^ On the other hand, another computational study^17^ has shown that the majority of backbone-NH order parameters in proteins fall into a relatively narrow range, which could preclude their usage for assessing changes in protein dynamics. Moreover, a recent experimental study has suggested that the main-chain contributions to configurational entropy change are relatively minor.^10^ In principle, one could use the above approaches to link configurational entropy with X-ray crystallographic B-factors (i.e., Debye–Waller or temperature factors)^48^ as well, since in the ideal case they also report on protein dynamics. We have previously shown that within the quasi-harmonic (QH) formalism,^25−27,32,35,49^ configurational entropy changes obtained exclusively from calculated B-factors (Δ*S*_Bfact_) display a strong linear correlation with the change in their QH value.^49^ However, since the QH approximation exhibits several major drawbacks^50^ (e.g., it assumes all potentials to be harmonic and accounts only for linear pairwise couplings, yielding values which are significantly higher than what is to be expected), the latter observation has to be further investigated.

Here, for the first time, we present a large-scale, self-consistent *in silico* comparison between the total MIST configurational entropy change (Δ*S*_MIST_) and the change in the average methyl order parameters (Δ⟨*O*_CH_3__^2^⟩), the average backbone-NH order parameters (Δ⟨*O*_NH_^2^⟩), or an entropy estimate derived from crystallographic B-factors (Δ*S*_Bfact_). The analysis is performed on a set of 19 1-μs-long molecular dynamics (MD) simulations of different proteins engaged in the formation of nine different binary complexes (also simulated for 1 μs). As free ubiquitin has been simulated five times and participates in the formation of four simulated complexes, this amounts to a total of 34 binding processes, i.e., transitions of a protein from a free to a bound state (Table 1, SI Figure 1).

**Table 1:** Simulated Protein Set: Molecule Names, Numbers of Atoms, PDB Codes, and Abbreviations

Name	No. atoms^a^	PDB code^b^	Complex^c^	Short name^d^	Abbreviation^e^
PPIase A	1641	1W8V	1AK4	PPIA	1
PR160Gag-Pol	1408	2PXR	1AK4	gag-pol	2
Alkaline protease	4503	1AKL	1JIW	aprA	3
Alkaline protease inhibitor	997	2RN4	1JIW	aprI	4
Subtilisin Carlsberg	2433	1SCD	1R0R	apr	5
Ovomucoid	498	2GKR	1R0R	OM	6
Uracil-DNA Glycosylase	2333	1AKZ	1UGH	UNG	7
Uracil-DNA Glycosylase inhibitor	788	1UGI	1UGH	UGI	8
Micronemal protein 6	496	2K2T	2K2S	MIC6	9
Micronemal protein 1	1226	2BVB	2K2S	MIC1	10
Tsg101 protein	1480	1KPP	1S1Q	TSG101	11
Ubiquitin	760	1UBQ	1S1Q	UBQ	12a,b,c,d,e^f^
ESCRT-I complex subunit VPS23	1493	3R3Q	1UZX	sst6	13
Ubiquitin	760	1UBQ	1UZX	UBQ	14a,b,c,d,e^f^
gGGA3 Gat domain^g^	949	1YD8*	1YD8	GGA3	15
Ubiquitin	760	1UBQ	1YD8	UBQ	16a,b,c,d,e^f^
E3 Ubiquitin-protein ligase CBL-B	457	2OOA	2OOB	CBLB	17
Ubiquitin	760	1UBQ	2OOB	UBQ	18a,b,c,d,e^f^

a Number of atoms in individual proteins.

b PDB codes^22,23^ of individual proteins.

c PDB codes of complexes.

d Short names used in the text.

e Key to abbreviations used in Figure 4a and SI Figure 3 and SI Figure 7.

f For ubiquitin, five separate simulations were used to generate the plots, reflected as the additional abbreviation tags a, b, c, d, and e.

g The constituent GGA3 Gat domain was extracted from the PDB structure of the 1YD8 complex and named 1YD8*, accordingly.

**Figure 1:**
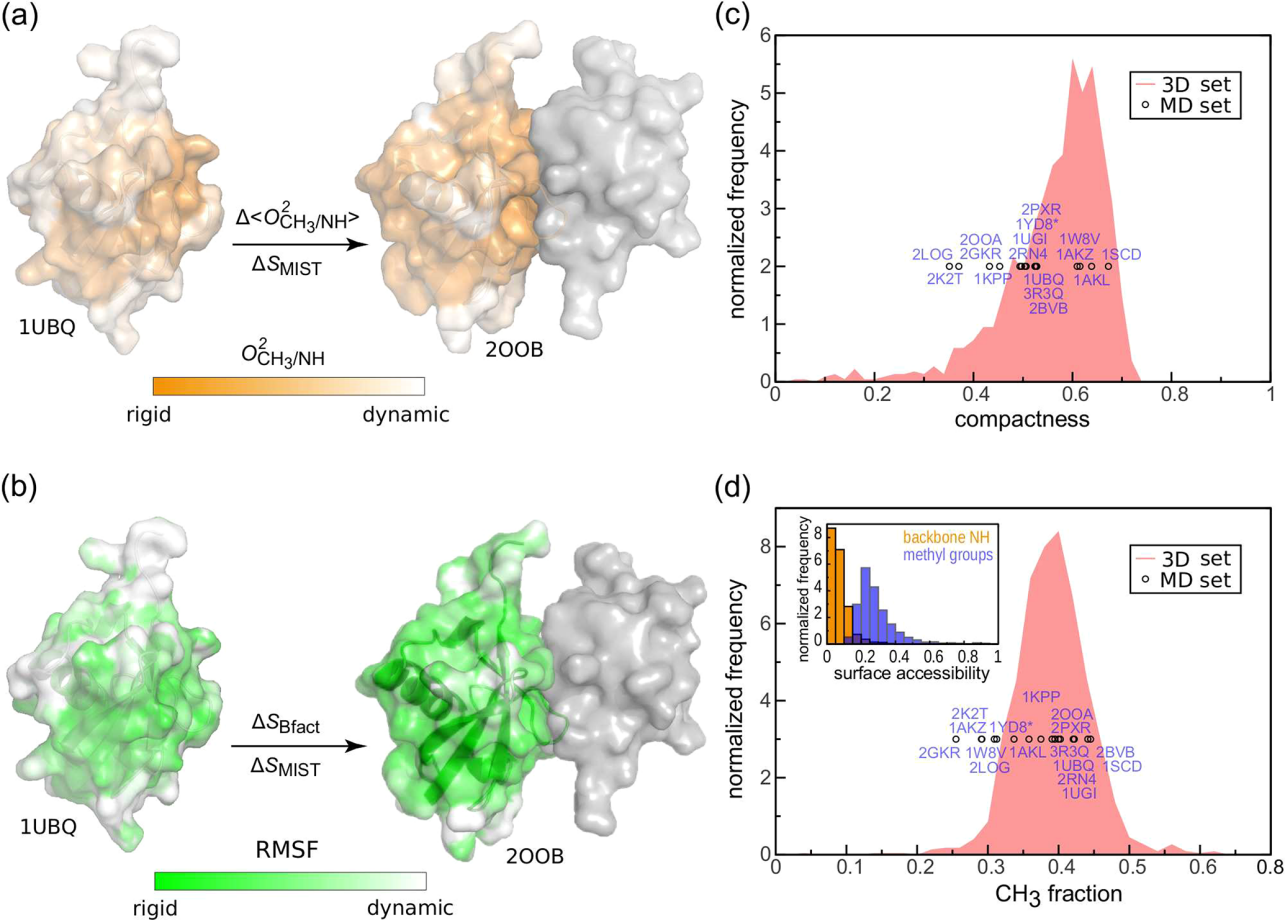
Self-consistent comparison of protein configurational entropy changes and experimental proxies of protein dynamics. For every protein, we independently calculate Δ*S*_MIST_ and (a) Δ⟨*O*_CH_3__^2^⟩ and Δ⟨*O*_NH_^2^⟩ or (b) Δ*S*_Bfact_ and correlate them against each other. (c) Compactness and (d) fraction of methyl-bearing residues for proteins used in this study as compared to the analogous values for the representative set of 1109 complete 3D structures^21^ from the PDB^22,23^ (red distributions).

**Figure 2:**
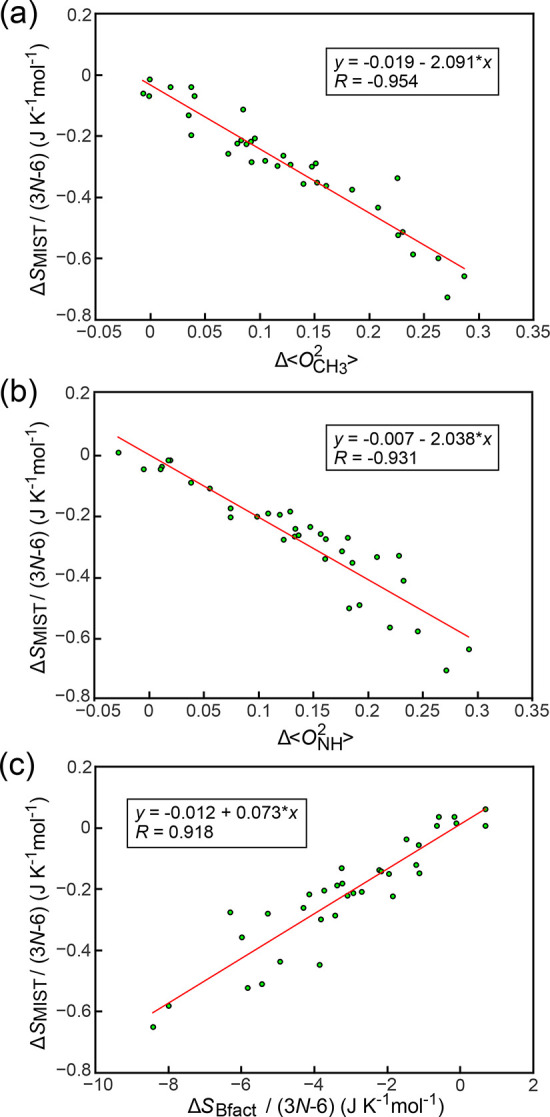
Comparison between experimentally accessible measures of protein dynamics and Δ*S*_MIST_. (a) Δ⟨*O*_CH_3__^2^⟩ vs Δ*S*_MIST_, (b) Δ⟨*O*_NH_^2^⟩ vs Δ*S*_MIST_, and (c) Δ*S*_Bfact_ vs Δ*S*_MIST_. All values reflect the entropy changes upon complex formation, evaluated separately for each individual protein. The Δ*S*_MIST_ and Δ*S*_Bfact_ values are normalized by the number of degrees of freedom in each protein (3*N* – 6, where *N* is the number of atoms). For each comparison, we provide the least-squares linear fit and the associated Pearson correlation coefficient *R*.

Here, we (1) provide an independent assessment of the validity of a linear connection between the change in the average NMR order parameters and the total configurational entropy change for realistic proteins [Figure 1(a)], (2) analyze different contributions to the total configurational entropy change, (3) expand the set of possible experimental observables to be used for protein configurational entropy estimation [Figure 1(b)], and (4) gauge the impact of undersampling on such estimation. Finally, with direct repercussions for these four aims, we evaluate the expected accuracy of experimental procedures based on linear relationships between the configurational entropy change and different experimental observables, with special attention to the impact of second-order couplings.

### 1.1 Physical Framework for Configurational Entropy Analysis

To embed configurational entropy into a physical framework, one starts from the following quasiclassical entropy integral:^51,52^
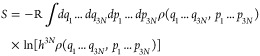
2

Here, R denotes the gas constant, *h* the Planck constant, *N* the number of atoms in the system, and ρ its phase-space probability density function (pdf), while *q*_*i*_ and *p*_*i*_ denote the spatial degrees of freedom and the canonically conjugate momenta in Cartesian coordinates, respectively. Assuming a Hamiltonian of the form
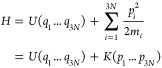
3where *m*_*i*_ denotes the (3-fold repetitive) mass vector of the system, and the pdf can be factorized into
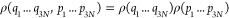
4

Then, eq 2 can be written in terms of spatial and momentum entropy^51^ as
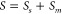
5with
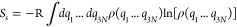
6and
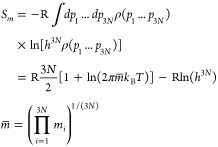
7

Here, *k*_*B*_ denotes the Boltzmann constant. For a molecule in solution and referring to the entropy from the degrees of freedom of the molecule only, the spatial entropy *S*_*s*_ is canonically termed configurational entropy. Note that the momentum entropy *S*_*m*_ in eq 7 is constant for fixed temperature and atomic composition.^51^ To further evaluate *S*_*s*_ in eq 6, one assumes a given concentration *C*° = 1/*V*° related to the corresponding container volume *V*^◦^ for a single molecule. If *U*(*q*_1_...*q*_3*N*_) in eq 3 is invariant under roto-translation, i.e., in the absence of any external field, the pdf can be divided into factors, which depend respectively on external and molecule-internal coordinates (such as anchored Cartesian^40,41^ or BAT^31,40−45^ coordinates) only. Then, integration over the six external degrees of freedom in eq 6 can be carried out analytically and *S*_*s*_ evaluates to^31,51,53^
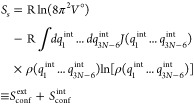
8with *J*(*q*_1_^int^...*q*_3*N*–6_^int^) being the part of the Jacobian dependent on the chosen internal degrees of freedom only. At a concentration of *C*° = 1/*V*° = 1 mol L^–3^, eq 8 defines the partial molar configurational entropy. As the first term in eq 8 is constant, the second term only is in literature frequently referred to as configurational entropy. Considering these definitions and assumptions, the following useful equation^39^ can be derived:^51^
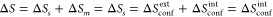
9

This equation explains that under the conditions as defined the total entropy change of a molecule is equal to its internal configurational entropy change, computable from the pdf of molecule-internal, spatial degrees of freedom only. Note that in the quasiclassical formalism upon separation of spatial and momentum degrees of freedom, as in (eqs 5–7), both quantities necessarily bear unphysical dimensions due to the form of the logarithmic term in eq 2. However, the problematic terms cancel for entropy changes,^51^ and thus, eq 9 is valid for entropy changes and for entropy changes only.

### 1.2 Introduction to the MIST Approximation

In contrast to the original derivation^29,36^ and for the sake of additional insight, we take a different approach here and introduce the MIST approximation as an optimum approximation to the MIE. If one defines generalized mutual information (MI) terms *I* as^31,54^
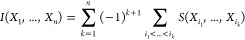
10one can express the entropy as
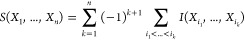
11

This expansion is known as the MIE. Note that for a single random variable *X*_*i*_ we have *I*(*X*_*i*_) = *S*(*X*_*i*_). Also note that, for example, a triplet MI term can be expressed using pairwise terms.^54^
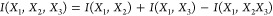
12

Note that the third term on the right-hand side of eq 12 constitutes a pairwise MI. It is defined as
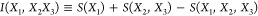
13

Pairwise MI is non-negative definite, and the following equation holds:^29,55^
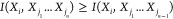
14

The use of the indices *j*_*k*_ here is intended to emphasize the arbitrariness of ordering. Harvesting the above equations, we can now introduce the principle behind the MIST approximation in the case of an entropy of three degrees of freedom
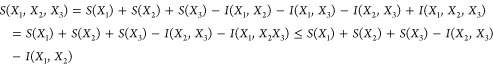
15

Note that there was a choice in the case of the last two MI terms, i.e., by performing the derivation slightly differently, one could have also chosen – *I*(*X*_1_,*X*_2_) – *I*(*X*_1_,*X*_3_) or – *I*(*X*_1_,*X*_3_) – *I*(*X*_2_,*X*_3_). As all of these choices constitute an upper bound to *S*(*X*_1_,*X*_2_,*X*_3_), the lowest upper bound is optimal. Thus, the choice in eq 15 was optimal if *I*(*X*_1_,*X*_3_) ≤ *I*(*X*_1_,*X*_2_) ∧ *I*(*X*_1_,*X*_3_) ≤ *I*(*X*_2_,*X*_3_). Applying the same principle consecutively, the MIST approximation for a higher order pdf can be expressed using marginal pdfs up to second order as^29,36^
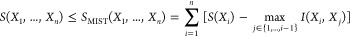
16

Operationally, this equation is implemented through the construction of the maximum spanning tree^56^ in order to identify the pairwise MI terms. Importantly, the MIST approximation is not restricted to configurational entropy^29,36^ and can be applied to arbitrary order, with each order mathematically guaranteed to yield a more accurate result. Finally, the approximation is guaranteed to provide an upper bound to the exact entropy value.

## 2 Results

### 2.1 Comparison of Changes in Configurational Entropy and Experimental Measures of Protein Dynamics

The simulated proteins and their complexes exhibit a variety in size and secondary and tertiary structures as well as biological function (Table 1, SI Figure 1). Additionally, they comprehensively cover the typical ranges of protein compactness [Figure 1(c)] and methyl-group abundance [Figure 1(d)]. In this sense, the simulated set can be seen as a representative sample of typical protein binding processes. Employing this set, we have correlated Δ⟨*O*_CH_3__^2^⟩, Δ⟨*O*_NH_^2^⟩ and Δ*S*_Bfact_ against Δ*S*_MIST_ representing the total configurational entropy change (Figure 2).

To account for artifacts resulting from the intrinsic difference in the abundance of experimental probes, Δ*S*_MIST_ and Δ*S*_Bfact_ are normalized by 3*N* – 6, where *N* is the number of simulated atoms in a given protein. Note that this is largely equivalent to the experimentally applied χ-angle normalization^10,12^ as both quantities are high-quality linear transformations of each other with a small offset. On the other hand, order parameters are averaged over all methyl and backbone-NH groups in the protein, as performed previously.^8,9^ The great majority (31/34) of binding processes in our set exhibit an increase in *O*_CH_3__^2^ upon binding, i.e., a decrease in the overall dynamics [Figure 2(a)], with only GGA3 (see Table 1 for abbreviations), aprA, and UNG becoming marginally more dynamic upon binding on average. More specifically, Δ⟨*O*_CH_3__^2^⟩ values span the range between approximately 0 and 0.3, with the proteins exhibiting the largest loss in dynamics being those with the highest dynamics prior to binding (SI Figure 2). The values of Δ*S*_MIST_ upon binding range between 0 and −0.7 J K^–1^ mol^–1^ per degree of freedom, corresponding to a substantial 500 kJ mol^–1^ of total free energy change at 300 K for the most extreme example (UGI forming a complex with UNG). In this case, the binding partner balances the drastic configurational entropy loss by increasing its own dynamics slightly (as can be seen from the *x*-axis in SI Figure 3 together with the legend in Table 1). Remarkably, Δ*S*_MIST_ per degree of freedom exhibits a strong linear relationship with Δ⟨*O*_CH_3__^2^⟩ with the absolute value of the Pearson correlation coefficient *R* between the two of 0.95 and no significant outliers over 34 binding processes [Figure 2(a), Δ*S*_MIST_ = (−2.09Δ⟨*O*_CH_3__^2^⟩ – 0.019)(3*N* – 6) J K^–1^ mol^–1^]. Notably, the value of the intercept of the linear fit between the two variables represents only a minor fraction of the complete range of fitted MIST values (0.019 vs −0.7 J K^–1^ mol^–1^, thus approximately 3%); to a good approximation, Δ*S*_MIST_ increases in direct proportion with the change in Δ⟨*O*_CH_3__^2^⟩. Importantly, the quality of this linear relationship is so good that it could, in principle, allow one to estimate Δ*S*_MIST_ directly from Δ⟨*O*_CH_3__^2^⟩. Below we analyze how quantitative such an estimation could be.

While methyl order parameters are considered to be good reporters of local dynamics because of the relative mobility of methyl groups,^12^ an advantage of backbone-NH order parameters is that they are present in every residue in the system. In contrast to the residue-specific inventory approaches,^57−59^ the results presented in Figure 2(b) support the utility of average backbone-NH order parameters for the estimation of configurational entropy. Namely, Δ⟨*O*_NH_^2^⟩ upon binding appears to be a quality measure of configurational entropy change, exhibiting a strong linear correlation with the MIST values [Δ*S*_MIST_ = (−2.04Δ⟨*O*_NH_^2^⟩ – 0.007)(3*N* – 6) J K^–1^ mol^–1^, Pearson *R* = −0.93] and a negligible *y*-axis intercept as compared to the range of the studied entropy change values [Figure 2(b)]. The interchangeability between the changes in the average methyl and NH-backbone parameters in this respect is certainly related to the fact that the two are closely related (Δ⟨*O*_CH_3__^2^⟩ = 0.91Δ⟨*O*_NH_^2^⟩ + 0.003, Pearson *R* = 0.91, SI Figure 4); the average change in side-chain dynamics upon binding appears to be an accurate predictor of the change in backbone dynamics and *vice versa*. Note that a previous study^17^ has indicated that this is the case only for backbone-NH order parameters below 0.8, and indeed, our values are well below this limit (SI Figure 2). While both methods accurately predict configurational entropy changes on their own, in the future, a combination of methyl and backbone-NH order parameters, as proposed in ref (17) but not excluding further proxies, may prove to be of significant value in fine-tuning experimental configurational entropy estimation. This also motivates the discussion of the results from the third, methodologically more distant, proxy parameter—the crystallographic B-factors.

Under an important assumption that B-factors report predominantly on local atomic motions, we have estimated their values for our simulated ensembles from atomic-positional root-mean-square fluctuations (RMSFs) after roto-translational superposition, according to a standard formalism (eq 20, see Section 5).^49,60^ Moreover, considering the nonlinear relationship between B-factors and configurational entropy, we have applied a QH approximation in Cartesian coordinates to calculate Δ*S*_Bfact_ values, as performed previously,^49^ before the comparison with the MIST values (eqs 18 and 19). Effectively, our configurational entropies derived from B-factors correspond to the Cartesian QH values in the absence of any intramolecular couplings; i.e., they capture the situation in which RMSF values report on the atomic positional variance and all covariance terms are set to zero. Remarkably, despite such a crude assumption, the derived configurational entropy change values correlate closely with the MIST values, obtained with a detailed treatment of anharmonicities and supralinear pairwise correlations in BAT coordinates [Figure 2(c)], with a Pearson *R* of 0.92 and only a handful of moderate outliers (UBQ binding to either CBLB or GGA3). As expected, the entropy changes derived from B-factors exceed the Δ*S*_MIST_ values by approximately 14-fold; i.e., correlations and anharmonicities captured by the MIST formalism together with the choice of the more decoupled BAT coordinate system reduce the overall configurational entropy changes by a factor of approximately 93% on the global scale across the full set of simulated proteins.

### 2.2 Robustness to Undersampling

**Figure 3:**
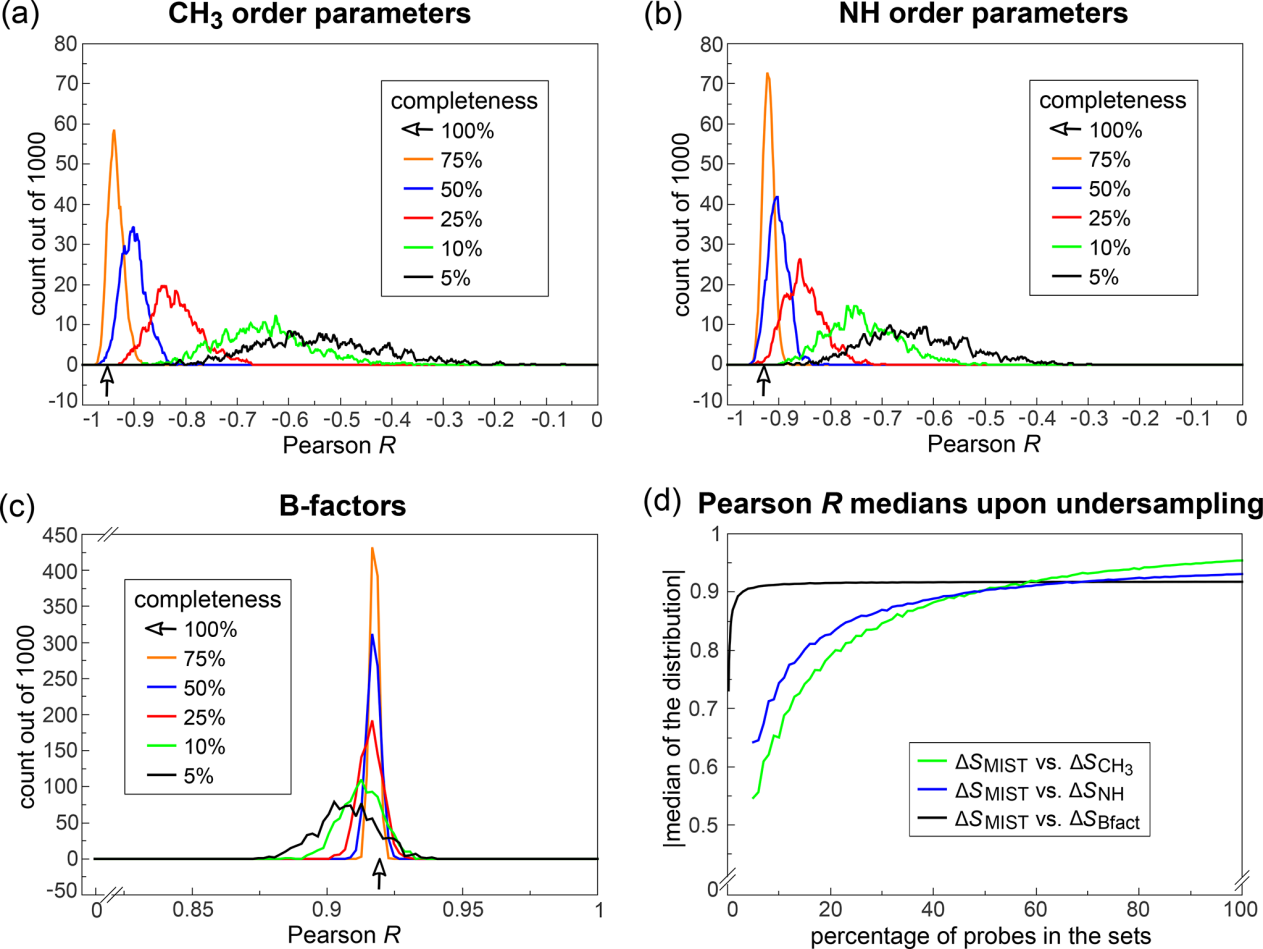
Dependence of the relationship between Δ*S*_MIST_ and different entropy proxies on the completeness of the set of experimental reporters. Distributions of Pearson correlation coefficients *R* between Δ*S*_MIST_, evaluated for the full set of degrees of freedom, and the undersampled (a) Δ⟨*O*_CH_3__^2^⟩, (b) Δ⟨*O*_NH_^2^⟩, or (c) Δ*S*_Bfact_ over the set of 34 binding processes. The degree of undersampling is given in the inset. Each distribution is based on 1000 independent repetitions of the undersampling procedure. All values are based on the changes upon complex formation, evaluated separately for each constituent and normalized by the number of degrees of freedom for Δ*S*_MIST_ and Δ*S*_Bfact_. The arrow marks the Pearson correlation *R* when taking the full set of reporters into account. (d) Absolute values of the medians of Pearson *R* histograms as a function of the degree of undersampling.

We have next analyzed the robustness of the above correlations against undersampling. The methyl order parameters, for example, are not distributed perfectly uniformly in proteins. While on average approximately 40% of residues contain a methyl group [Figure 1(d)], their total abundance changes from protein to protein, and they are, in general, difficult to measure to completeness. It is, therefore, of direct practical significance to know how large a subset of reporters of a given type is needed to still provide useful information on the overall configurational entropy change. To address this challenge, we have selected at random a subset of a given size of the experimental reporters in question and then correlated the configurational entropy changes derived from these probes with the MIST values calculated using all degrees of freedom. By iterating this procedure 1000 times, we have obtained 1000 Pearson *R* values reporting on the overall impact of undersampling on configurational entropy change estimation. In Figure 3, we show the distributions of Pearson *R* values obtained at different levels of undersampling for the three types of experimental probes in question.

Remarkably, the above correlations appear to be extremely robust against undersampling. In the case of both methyl and backbone-NH order parameters [Figure 3(a) and (b)], a correlation against the Δ*S*_MIST_ values with the absolute value of Pearson *R* at 0.9 is obtained already if one includes only 50% of the total number of all available reporters (methyl and backbone-NH order parameters, respectively) in each protein. Even more impressively, by only selecting at random 5% of the atoms in every protein, one is practically guaranteed to obtain a correlation between Δ*S*_Bfact_ and the full Δ*S*_MIST_ of approximately 0.9 or higher [Figure 3(c)]. Interestingly, at approximately 60% of all reporters of a given type or higher, the order parameter-based approaches outperform slightly the equivalent B-factor-based approach [Figure 3(d)]. One should, however, remember that the order parameters even in the ideal case report on a subsample of the total degrees of freedom only. Thus, a direct comparison of the effects of undersampling in the case of order parameters and B-factors may *a priori* be biased. On the other hand, given that the number of atoms in a typical protein is approximately 17 times larger than the number of methyl order parameters, the order parameters appear to provide a considerably higher quality measure of configurational entropy changes as compared to crystallographic B-factors on an absolute per reporter basis. It should be noted, however, that for NMR order parameters, the distribution of slopes changes significantly upon undersampling [SI Figure 5(a) and SI Figure 5(b)], which can be seen from the width of the distributions. Also, the slopes are systematically biased by the number of probes, i.e., the measured order parameters. This is explained by the fact that the slope of a linear fit can mathematically be expressed as the covariance divided by the variance, two variables which are essentially proportional to the inverse and the squared inverse of the number of probes, respectively. Thus, in the case of undersampling, the magnitude of calculated slopes will be biased toward lower values. For crystallographic B-factors, the situation is drastically improved [SI Figure 5(c)], simply by the virtue of their abundance.

### 2.3 Expected Experimental Accuracy

**Figure 4:**
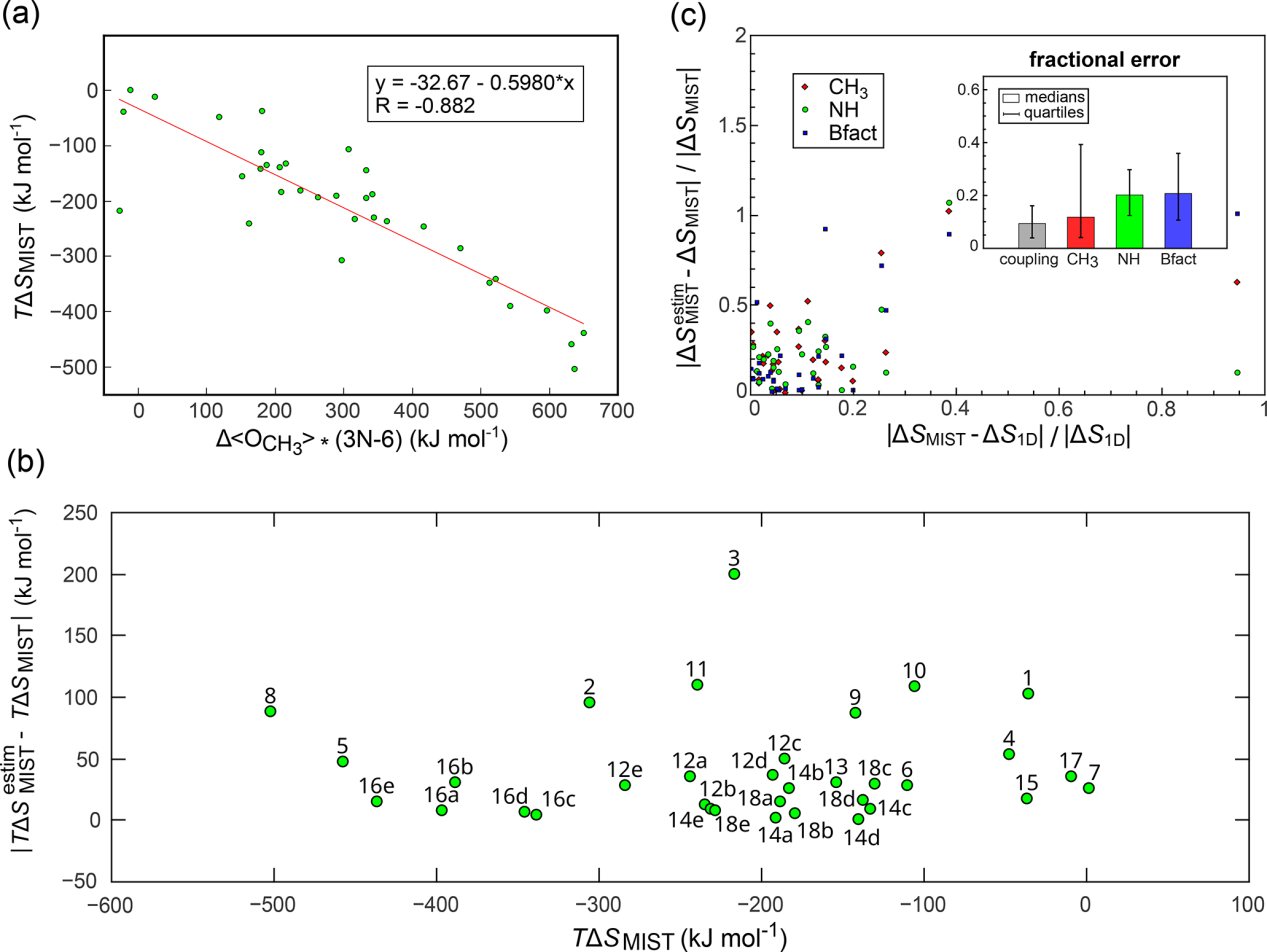
Error analysis. (a) Comparison of un-normalized Δ*S*_MIST_ against the average methyl order parameters scaled by the respective number of degrees of freedom. (b) Errors as the absolute value of the deviation along the *y*-axis from the linear regression in (a). (c) Relationship between fractional coupling, |Δ*S*_MIST_–Δ*S*_1D_|/|Δ*S*_1D_|, and fractional error, |Δ*S*_MIST_^estim^ – Δ*S*_MIST_|/|Δ*S*_MIST_|, for all three experimental probes. For clarity, the range of the graph has been truncated to show 91% of all of the data. The remaining outliers stem from vanishing denominators on both axes. The inset shows the medians and the quartiles of fractional couplings and fractional errors for all three experimental probes. In panel (c), errors were estimated based on the linear regressions given in Figure 2.

The above results demonstrate a remarkably strong linear relationship between different experimental proxies and Δ*S*_MIST_ normalized by the number of degrees of freedom. However, in order to evaluate the applicability of such linear relationships for deriving the configurational entropy change in an experimental context, one needs to assess the accuracy of the derived absolute configurational entropy changes. Figure 4(a) shows the analogue of Figure 2(a) for absolute entropy changes as obtained by scaling the average methyl order parameters with the respective number of degrees of freedom, thereby rendering the entropy changes an extensive quantity. The respective absolute error, i.e., the absolute *y*-axis deviation from the line of the linear fit, is shown in Figure 4(b). As expected, Figure 4(a) demonstrates a similarly high correlation as its intensive analogue Figure 2(a). Importantly, however, the errors are large in absolute terms and do not scale with the magnitude of Δ*S*_MIST_. At a temperature of 300 K, the root-mean-square error over all simulated binding processes amounts to a significant value 59.5 kJ/mol. A similar situation is observed for backbone-NH order parameters and crystallographic B-factors as well (SI Figure 6 and SI Figure 7), with the root-mean-square errors of 57.3 and 56.1 kJ/mol, respectively. Taking this at face value, these results suggest that regardless of the degree to which dynamics of a protein changes upon binding or the choice of one of the three experimental proxies discussed, an error of about 60 kJ/mol is to be expected. Importantly, this value is based on an idealized, self-consistent *in silico* analysis in which one has control over all of the degrees of freedom and the dynamics of the protein is fully known. It is likely that any analogous experimental procedure would be associated with even greater errors.

Intuitively, the most likely source of such large errors could reside in the couplings between different degrees of freedom, and this is why we have analyzed these terms in greater detail. Reverting back to the estimates based on the linear fits given in Figure 2, in Figure 4(c) we show the relationship between the fractional error in the configurational entropy estimates |Δ*S*_MIST_^estim^ – Δ*S*_MIST_|/|Δ*S*_MIST_| and the fractional contribution of coupling terms to the absolute configurational entropy changes |Δ*S*_MIST_–Δ*S*_1D_|/|Δ*S*_1D_|, where Δ*S*_1D_ denotes the configurational entropy change if one neglects all couplings (defined as mutual information here). In general, the values of both the fractional error and the fractional coupling are at or below ≈0.2 for the majority of the cases (see below for details). Moreover, there is a general trend in that large errors, and therefore large deviations from linearity, are more common for systems that exhibit a large change in couplings as compared to Δ*S*_1D_. This trend seems to be valid for all three experimental probes. The Figure 4 inset shows the median value over all studied systems for the fractional coupling (9%) as well as the median fractional errors for the estimates based on methyl order parameters (12%), NH order parameters (20%), and B-factors (21%). Errors normalized per degree of freedom are shown in SI Figure 3. Overall, the average values of |Δ*S*_MIST_^estim^ – Δ*S*_MIST_|/(3*N* – 6) across all simulated systems are 0.04, 0.052, and 0.056 J K^–1^ mol^–1^ per degree of freedom for methyl, NH order parameters, and B-factors, respectively. The average value of Δ*S*_MIST_/(3*N* – 6) over all simulated systems is 0.275 J K^–1^ mol^–1^ per degree of freedom. At this point, it is important to note that the absolute errors, which are indeed sizable, still translate to relatively moderate fractional errors in the contribution of the configurational entropy change to the binding free energy change (≈10%–20%). Namely, entropic and enthalpic free-energy terms usually represent large quantities, which can strongly compensate each other and result in the total binding free energy change that is less than the aforementioned absolute errors.

### 2.4 Comparison of Coupling

**Figure 5:**
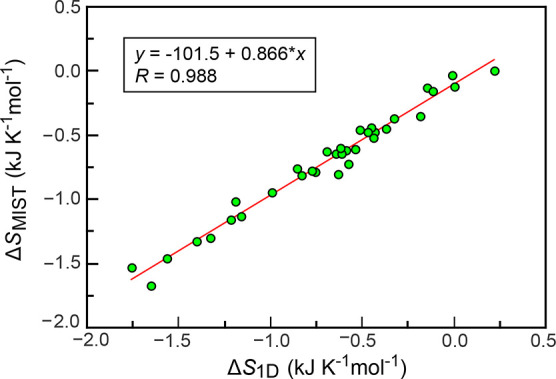
Effect of pairwise couplings in the MIST approximation. Shown are configurational entropy changes upon binding for every protein in the simulated set, whereby coupling corrections of pairwise order are included on the *y*-axis and excluded on the *x*-axis. The values are normalized by the number of degrees of freedom of the respective molecules.

As discussed above, a major issue in configurational entropy estimation concerns the degree to which coupled motions reduce the overall entropy values. In agreement with our previous results,^49^ the pairwise, linear intramolecular couplings, as estimated within the framework of the QH approximation in Cartesian coordinates on the present set, reduce the configurational entropy changes by an approximately constant fraction of 80% (SI Figure 8). In other words, configurational entropy changes obtained if one ignores the covariance terms within the QH approximation (i.e., Δ*S*_Bfact_) correlate linearly with the QH values obtained by treating the covariances fully (Δ*S*_QH_), exhibiting a slope of approximately 0.2 (SI Figure 8, Δ*S*_QH_ = 0.21Δ*S*_Bfact_ – 814.9 J K^–1^ mol^–1^, Pearson *R* = 0.89). On the other hand, in the case of the MIST approximation in BAT coordinates, the inclusion of correlation terms results in a much smaller correction to the uncorrelated configurational entropy changes (Figure 5, Δ*S*_MIST_ = 0.87Δ*S*_1D_–101.5 J K^–1^ mol^–1^, Pearson *R* = 0.99), i.e., a reduction of approximately only 13% on average. This, in general, suggests that the contribution of coupling may be surprisingly predictable, although individual proteins could deviate from the typical behavior, as further discussed below. Note, however, that in 53% of our simulated systems coupling leads to a decrease in the uncoupled configurational entropy changes (which are negative apart from PPIase A) as opposed to an increase (in the case of absolute values, coupled motions necessarily always decrease the entropy). Such a diverse behavior was observed previously in several case studies.^30,38^ We find that for binding processes involving ubiquitin in particular, coupling contributions tend to predominantly increase the value of the total configurational entropy change (65% of cases), thus counteracting the uncoupled entropy changes. Other proteins exhibit the opposite tendency (79% of cases decreasing). However, the magnitude of increasing contributions is commonly larger, with only 47% of all cases exhibiting an increase, yet the average reduction of the magnitudes being 13% across the whole set (Figure 5).

## 3 Discussion

The results presented herein provide a quantitative, self-consistent foundation for exploiting NMR order parameters and X-ray crystallographic B-factors for the determination of configurational entropy changes in proteins. Specifically, our results suggest that total configurational entropy changes calculated within the MIST framework in BAT coordinates, which takes into the account anharmonicity of the potentials and linear and supralinear couplings up to pairwise order and avoids spurious correlations induced by the usage of Cartesian coordinates,^61^ exhibit a rather robust linear relationship with the changes in the average NMR order parameters or the quasiharmonic configurational entropy changes derived from B-factors. What makes this finding particularly remarkable is that the three experimentally derivable proxies of configurational entropy change analyzed here include no direct information on intramolecular couplings. We would like to suggest that this robustness could be an intrinsic property of compact proteins whereby the degree to which configurational entropy change is reduced due to pairwise intramolecular couplings is on average directly proportional to the entropy change due to the leading, uncoupled terms. Indeed, our analysis(Figure 5) demonstrates that the pairwise linear couplings in the case of the QH approximation or full pairwise couplings in the case of the MIST approximation reduce the respective Δ*S* values by on average ≈79% in the QH and ≈13% in the MIST approximation, yielding a high-quality correlation between the uncoupled and the coupling-corrected values. While the full QH changes are generally higher than the full MIST changes by ≈3 fold (SI Figure 9), the fractional difference of coupling stems arguably from the different coordinate systems used, i.e., is due to the spurious correlations in Cartesians in the case of the QH approximation.^61^

**Figure 6:**
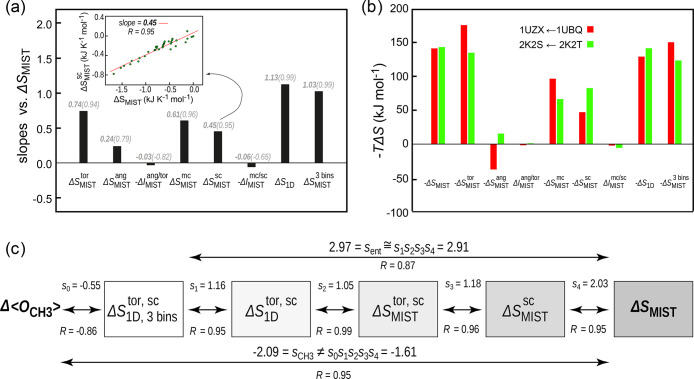
Contributions to the total configurational entropy change. (a) Average contributions across the whole protein set. Shown are the magnitudes of the change in torsional (Δ*S*_MIST_^tor^), and angular (Δ*S*_MIST_^ang^) entropy contributions and their mutual coupling (−Δ*I*_MIST_^ang/tor^) and main-chain (Δ*S*_MIST_^mc^) and side-chain (Δ*S*_MIST_^sc^) contributions and their mutual coupling (Δ*S*_MIST_^mc/sc^) as well as uncoupled entropy change (Δ*S*_1D_) and total configurational MIST entropy with vibrations excluded by coarse-graining the sampled probability distributions to three bins only (Δ*S*_MIST_^3 bins^). The bars represent the value of the slope of the linear fit between the contributions in question and the total configurational entropy change, while the values in parentheses indicate the associated Pearson *R*s. The fitting procedure is illustrated for the case of the side-chain contribution in the inset. (b) Absolute values of different entropic terms including temperature and no normalization for two different binding processes. (c) High-quality linear relationships involved allow one to use the slopes of individual steps in order to estimate the full configurational entropy change Δ*S*_MIST_ (top arrow) starting from the vibration-suppressed, uncoupled torsional side-chain entropy, which is directly approximated by NMR methyl order parameters. However, starting from the order parameter changes, such transitivity is broken.

Further mechanistic insight concerning the above linear relationships can be obtained by dissecting the total configurational entropy change into its main components. In Figure 6(a), we show the results of such a dissection when it comes to torsional and angular contributions and their mutual coupling as well as main-chain and side-chain contributions and their mutual coupling. We also show the contributions of the uncoupled configurational entropy and the total configurational MIST entropy with vibrations excluded by coarse-graining the sampled probability distributions to three bins only. As demonstrated in the case of side-chain contributions [Figure 6(a) inset], the relative magnitude of the contributing terms was estimated from the slope of a linear fit against the total configurational entropy change. Remarkably, all of the above components exhibit strong linear relationships against the total configurational entropy change as evidenced by the high associated Pearson correlation coefficients. This is especially true for the strongly contributing terms, which all exhibit Pearson correlation coefficients ≥0.94. Overall, the coupling contributions between torsional and angular terms as well as those between main-chain and side-chain terms are rather insignificant. On the other hand, our results strongly suggest that the angular terms contribute ≈24% of the total configurational entropy change and are thus far from negligible. This is qualitatively supported by the findings of Gilson and co-workers in the case of binding between the ubiquitin E2 variant domain of the protein Tsg101 and an HIV-derived nonapeptide.^30^ However, as compared to the latter case study performed on an individual system, our results provide a general statement across a large set of different binding processes [Figure 6(a)].

As illustrated in Figure 6(b), our results also demonstrate that different proteins may exhibit significantly different behavior in this regard. While the two proteins shown in Figure 6(b) exhibit almost the same total configurational entropy change upon binding, its dominant components, namely, those belonging to torsional, angular, and main-chain and side-chain degrees of freedom, deviate by around 30 to 50 kJ mol^–1^. Interestingly, for these particular binding processes, the conformational components with the vibrational contribution removed also deviate by 37 kJ mol^–1^. However, considering the low impact of removing vibrations, as shown in Figure 6(a), together with the high correlation observed, this clearly is not a general case but rather an isolated case. In order to remove the vibrational contributions, configurational probability distributions were coarse-grained by using only three bins in our entropy calculations (see the Methods Section for further discussion). A more rigorous separation^20,62^ of conformational entropy from vibrational entropy is technically impractical given the large-scale nature of the present analysis (there is a fundamental arbitrariness in such a decomposition^53,62^ in any case). Also note that in the case of ubiquitin binding one can even observe a different sign in angular contributions depending on the nature of its binding partner. When it comes to main-chain contributions, our findings are in accordance with a recent computational study on bovine pancreatic trypsin inhibitor,^38^ but they may appear to be at odds with the recent findings by Wand and co-workers.^10,12,17^ However, the numerical analysis provided in ref (17) suggests that the average NH-backbone order parameters correlate well with the average side-chain methyl order parameters if their value is below ≈0.8. As this is the case for all of our proteins (SI Figure 2) and we indeed do observe a high quality correlation (SI Figure 4), there is no contradiction. A follow-up experimental investigation^10^ from the same group suggested that the backbone contributions to configurational entropy may be small, although difficulties in measurement have been reported. Moreover, these studies have also suggested that the angular contributions are small, but our analysis shows not only that these contributions scales linearly with the total configurational entropy change (as does the main-chain contribution) but also that they are too large to be ignored.

Overall, our study supports the usage of the change in the average methyl order parameters for estimating the configurational entropy change by using a semiempirical fitting procedure, as championed by Wand and others^7−10,12^ although, as discussed below, the associated errors may be prohibitively high. Importantly, although there exist terms which are not directly probed by the side-chain methyl order parameters, they are already indirectly fully accounted for by the applied linear fit, which is the key element of the proposed ”entropy meter”. In other words, the above linear relationships between the total configurational entropy change and its different components allow one to estimate the total configurational entropy change from just a subset of the contributing degrees of freedom, such as the side-chain torsional, vibration-less contributions probed by the experiment. This important notion is supported further by the results presented in Figure 6(c). There, we demonstrate that by including successively more and more contributions to the configurational entropy estimate, one obtains almost the same slope as if directly calculating the slope between the uncoupled, vibration-suppressed, side-chain torsional entropy change and the total configurational entropy change. Crucially, this transitivity of slopes is enabled by a high-quality linear relationship at every step. Interestingly, the methyl order parameters, however, report significantly better on the total configurational entropy change than on the uncoupled, vibration-suppressed, side-chain torsional entropy change.

It is likely, however, that the above linearities hold only for proteins with a similar level of moderate compactness, such as those in our present set [Figure 1(c)]. In order to address this possibility, we have further investigated the binding between ubiquitin and the highly dynamic UBM2 protein which was previously shown to be of a significantly lower compactness as compared to other proteins in our set.^49^ Five simulations were run for both the UBM2 unbound state and the complex with ubiquitin, yielding a total of 25 different values of Δ*S*_MIST_. Indeed, as illustrated in SI Figure 10, we see a noticeable drop in the quality of the linear fit for all three dynamics measures as compared to Figure 2 and a marked change in slope in the case of NH order parameters and B-factors. The high-quality correlations are retained for all three dynamics proxies in the case of the second binding partner ubiquitin, a compact, well-folded protein (SI Figure 11), although the slopes for the NH order parameter and the B-factor plots change in comparison to those in Figure 2. This suggests that for increased precision the linear relationships between Δ*S* and dynamic proxies should be calibrated on a specific set of interest, as done previously.^8,9^ Interestingly, however, when comparing to the recent experimental results obtained for calmodulin binding to different helical peptides,^10^ whereby both calorimetric entropy values, corrected for solvent contributions, as well as methyl order parameters were determined, the computational slope we fit in Figure 2(a) comes to within 20% from the experimentally fitted value (SI Figure 12).

A previous computational study suggested the use crystallographic B-factors for the estimation of configurational entropy changes.^49^ Here, using the advanced MIST approximation, we are able to eliminate concerns about both the deficiencies of the QH approach^50^ and the potential bias introduced by using the same method for the prediction of coupled and decoupled configurational entropy as was done previously. However, it should also be stressed that B-factors are influenced by issues such as rigid-body motions, crystal imperfections, and refinement artifacts.^63^

Last but not least, it should be emphasized here that despite the high-quality linear relationships presented throughout this study, the magnitude of the expected experimental error is significantly large for all three experimental proxies discussed. At 300 K, one can expect an error of about 60 kJ/mol, which is surprisingly independent of the magnitude of Δ*S*_MIST_. What is more, this estimate should be considered to be a lower bound on the errors one might see in an experiment, as here our estimate is based on an idealized test case where one has control over all degrees of freedom and the dynamics is known exactly. In other words, in our computational analysis, the simulated trajectories are fixed, and entropies as well as all the proxy parameters are calculated self-consistently from the same simulated configurations. In this context, it would be interesting to see whether one of the entropy components analyzed in Figure 6(a) could be mainly responsible for the expected error. Unfortunately, correlating the deviation from linearity as in the inset of Figure 6(a) against the error for methyl order parameters in Figure 4(a) did not give any conclusive results (see Table 2). Here, the Pearson *R* gives a measure of the quality of the impact of a given contribution on the expected measurement error, while the slope gives a measure of the magnitude of the impact. Because of relatively low values of Pearson *R*s, however, it appears that the error cannot be directly attributed to any specific contribution. Finally, the situation is similar for backbone-NH order parameters and even worse for B-factors (data not shown). Nevertheless, although the expected accuracy of this recalibration method restricts its usage to applications where a more qualitative rather than a precise value is sufficient, its pioneering nature has enabled basic experimental access to the thermodynamic measure of the extent of protein dynamics, namely, configurational entropy.

**Table 2:** Effect of Different Configurational Entropy Contributions on Estimated Error for Methyl Order Parameters

Quantity	Pearson *R*	slope
Δ*S*_MIST_^tor^	–0.33	–0.59
Δ*S*_MIST_^ang^	–0.01	–0.03
Δ*S*_MIST_^ang/tor^	0.33	6.63
Δ*S*_MIST_^mc^	0.12	0.34
Δ*S*_MIST_^sc^	–0.14	–0.47
Δ*S*_MIST_^mc/sc^	–0.02	–0.13
Δ*S*_1*D*_	–0.21	–0.57
Δ*S*_MIST_^3 bins^	–0.28	–0.76

## 4 Conclusions

By employing our newly developed parallel program suite,^39^ we have carried out the largest-yet computational study of configurational entropy using an advanced state-of-the-art information theoretical method. Our results support the pioneering NMR approaches^8,9,12,64^ for the determination of configurational entropy from methyl order parameters, but the expected accuracy of estimates obtained in such a way restricts their usage to applications where qualitative analysis may be sufficient. We have demonstrated that even in such cases it is crucial to apply recalibration, as such a procedure naturally includes coupling corrections as well as contributions from the main-chain and angular degrees of freedom, which may be significant. The reason that such recalibration can at all be successful is a high-quality, unexpected linear relationship between the full configurational entropy change and the uncoupled side-chain torsional rotamer entropy change, directly proxied by NMR methyl order parameters. Furthermore, the set of experimental observables was expanded by the NMR NH backbone order parameters and, remarkably, crystallographic B-factors. We hope that the present work will contribute to a more widespread development and application of experimental methods for configurational entropy estimation in proteins. We are convinced that such efforts will contribute to a deeper understanding of configurational entropy in fundamental and practical contexts alike.

## 5 Methods

### 5.1 Molecular Dynamics Simulations

MD simulations were performed as described previously^39,49^ using the GROMACS 4.0.7 simulation package,^65,66^ the GROMOS 45A3 force field,^67^ and the SPC water model.^68^ Proteins were placed in water boxes, together with the necessary number of sodium or chloride counterions to reach neutrality, and subjected to energy minimization, followed by heating to 300 K for 100 ps and subsequent unconstrained MD simulations. The length of each MD trajectory was 1 μs, with the first 200 ns treated as an equilibration period and the remaining 800 ns analyzed. Simulations were carried out with a time step of 2 fs using 3D periodic boundary conditions, in the isothermal–isobaric (NPT) ensemble with an isotropic pressure of 1 bar and a constant temperature of 300 K, while system coordinates were output every 1 ps. The pressure and the temperature were controlled using the Berendsen thermostat and barostat^69^ with 1.0 and 0.1 ps relaxation parameters, respectively, and a compressibility of 4.5 × 10^–5^ bar^–1^ for the barostat. Bond lengths were constrained using LINCS.^70^ The van der Waals interactions were treated using a cutoff of 14 Å. Electrostatic interactions were evaluated using the reaction-field method,^71^ with a direct sum cutoff of 14 Å and relative permittivity of 61.

The PDB codes^22,23^ of the simulated complexes and their constituents are 1UBQ, 1S1Q, 1KPP, 1UZX, 3R3Q, 1YD8, 2OOB, 2OOA, 1AK4, 2PXR, 1W8V, 1JIW, 2RN4, 1AKL, 1R0R, 2GKR, 1SCD, 1UGH, 1UGI, 1AKZ, 2K2S, 2K2T, and 2BVB. Note that for 1UBQ (ubiquitin), five separate simulations were run. Furthermore, note that 1UBQ is a constituent of the complexes 1S1Q, 1UZX, 1YD8, and 2OOB. For the complex 1YD8, due to the lack of a separate structure, the ubiquitin binding partner (human GGA3 GAT domain) was extracted from the PDB structure of the complex and equilibrated for an additional 500 ns. Further details are given in Table 1.

Protein compactness was estimated as the ratio between the protein solvent-accessible surface in the folded structure and in the fully elongated structure.^49^

### 5.2 Maximum Information Spanning Tree (MIST) Entropy Calculations

The configurational entropy was evaluated by applying the MIST approximation.^29,36^ Entropy calculations were carried out using the PARENT^39^ suite, a collection of programs for the computation-intensive estimation of configurational entropy by information theoretical approaches using a parallel architecture. First, the trajectories were converted from Cartesian to BAT coordinates.^31,41−45^ The PARENT core program was then employed in order to yield 1D entropy values for all degrees of freedom in a given system as well as 2D entropy values for all pairwise combinations of degrees of freedom. Couplings of an order higher than pairwise were neglected for reasons of computational feasibility. For sampling probability densities, 50 bins were used in one-dimensional cases and 50 × 50 = 2500 in two-dimensional cases. Using the obtained entropy terms, the PARENT program suite was employed to apply the MIST approximation^29,36^ to the full set of degrees of freedom by constructing the maximum information spanning tree.

### 5.3 NMR Order Parameters

Adopting the “model-free” formalism of Lipari and Szabo,^11^ generalized NMR order parameters were extracted from MD trajectories using the formula^72−74^
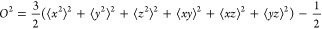
17

Here, *x*, *y*, and *z* are the Cartesian coordinates of the bond vector associated to the order parameter in unit length. The overall tumbling (rigid-body rotation and translation) of the molecule was separated from internal dynamics by subjecting solute conformers to a least-squares superposition of all atoms (the same for RMSF calculations).

### 5.4 Crystallographic B-Factor Entropy Calculations

For the calculation of entropy values derived from B-factors, a previously described procedure^49^ was applied. Atom-positional Cartesian root-mean-square fluctuations (RMSFs) of all the atoms in a given protein or complex were obtained by using the program g_covar of the GROMACS^65,66^ simulation package, with all nondiagonal elements of the variance-covariance matrix discarded. The diagonal elements, representing the atomic *x*-, *y*-, and *z*-RMSF values, were then inserted into the formula^26,35,49^
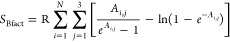
18where
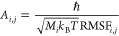
19to obtain the configurational entropy neglecting all couplings in the system. Here, R denotes the gas constant, *k*_*B*_ the Boltzmann constant, *ℏ* the reduced Planck constant, *M*_*i*_ the mass of the atom *i*, *T* the system temperature, and RMSF_*i*,*j*_ is the root-mean-square fluctuation of the Cartesian coordinate *j* belonging to atom *i*. It is calculated from the MD trajectory as
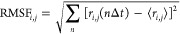
20where *r*_*i*,*j*_ denotes the Cartesian coordinate *j* from atom *i*, and *n*Δ*t* is the timestamp of the *n*th discrete frame in the MD trajectory. The brackets ⟨⟩ denote averages over the whole trajectory. For the calculation of configurational entropy values from experimental B-factors, the relationships^49^
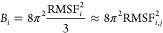
21thus
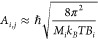
22and
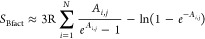
23were used. Here, *B*_*i*_ denotes the B-factor of atom *i*. The other variables and constants are the same as in eq 18.

### 5.5 Vibrational Entropy Estimate

For a set of discrete states, their entropy, in general, changes if one introduces new states of nonvanishing probability to the system. For example, the entropy of a uniform distribution strictly increases with the number of states (Shannon’s second Axiom^75^). If, by analogy, the number of bins of a discretized continuous probability distribution (yielding discretized differential entropy^28,51^) is increased, the entropy value is affected only if the fine-grained probability distribution reveals additional features. To elaborate on this property, the formula for the discretized differential entropy is stated^28^
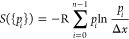
24

This expression differs from the entropy of a set of discrete states by the inclusion of the inverse of the bin size Δ*x* inside the logarithm. Here, *p*_*i*_ denotes the probability of the system to be found inside bin *i*, *n* marks the total number of bins, and the curvy brackets denote the set of all *p*_*i*_. R denotes the gas constant. Now, in order to increase the resolution of discretization without adding new features to the probability distribution, we define the following derived probability distribution, which features additional fine-graining by a factor *f*.
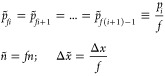
25Then
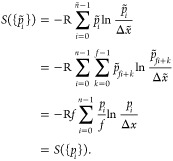
26

In other words, an increase in the degree of graininess does not affect the value of entropy if no additional features are introduced. Note that, for simplicity of notation, we have neglected a Jacobian contribution here, which due to nonlinearity could, in principle, affect the above result. The Jacobian in BAT coordinates reads^28,31,51^
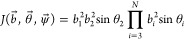
27where *b* denotes bond lengths, θ angles between bonds and ψ torsional angles, and *N* the number of atoms in the molecule. Note that torsional angles do not contribute to the Jacobian, although they constitute ≈74% of the configurational entropy [Figure 6(a)]. Furthermore, bonds and angles between bonds are rather stiff degrees of freedom, and additionally, the angles enter the Jacobian inside a sine function. Thus the Jacobian hardly adds a contribution to single molecule entropy changes for most proteins.^31^ Another consideration is that the usage of three bins will often split conformers apart, thus yielding a poor estimate for conformational entropy. While this holds true, small amplitude motions, i.e., vibrations, will still mostly be efficiently suppressed. Thus, while a decrease in the number of bins will not necessarily give a precise estimate of conformational contributions, we expect it to serve well for demonstrating in a qualitative manner that fast vibrational motions hardly contribute to configurational entropy changes associated with protein–protein complex formation.

## Supporting Information Available

List of the proteins used in the present study; figure demonstrating the impact of the overall dynamics before binding on the change of dynamics upon binding; error analysis for estimating the MIST entropy changes using slopes and offsets only, both with and without normalization by the number of degrees of freedom; graph relating the methyl to the backbone-NH order parameter changes; graph relating the proxy quantities to configurational entropy without normalization; analysis of the effect of undersampling on the fitted slopes; analysis of the coupling in the quasi-harmonic approximation; graph relating MIST to quasi-harmonic entropy; two figures elaborating on UBQ binding to a low compact binding motif (UBM2); and analysis of recent experimental data. (PDF)
