# Supplementary material for: Self-Consistent Framework Connecting Experimental Proxies of Protein Dynamics with Configurational Entropy
Source: J Chem Theory Comput. 2018 May 25;14(7):3796–810. doi: 10.1021/acs.jctc.8b00100 (PMC9245193; doi:10.1021/acs.jctc.8b00100)
Supplement: Supplementary file 1 — ct8b00100_si_001.pdf [file ct8b00100_si_001.pdf]

Supplementary information: Self-Consistent Framework Connecting Experimental Proxies of Protein Dynamics with Configurational Entropy

Markus Fleck, Anton A. Polyansky, and Bojan Zagrovic*

Department of Structural and Computational Biology, Max F. Perutz Laboratories, University of Vienna, Campus Vienna Biocenter 5, Vienna, 1030, Austria.

E-mail: bojan.zagrovic@univie.ac.at

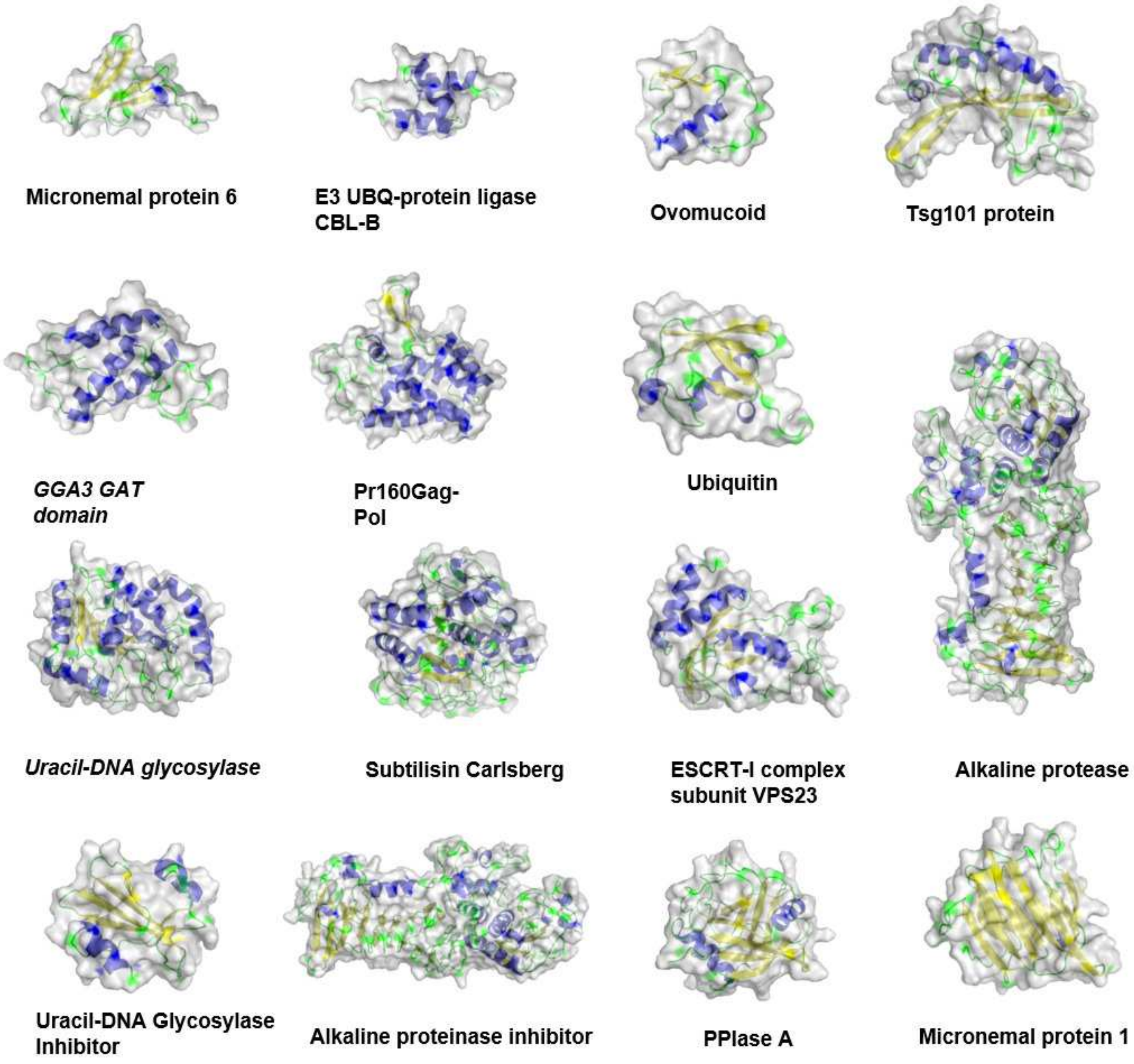

Figure 1: Cartoon representation of the secondary structure of proteins used in the study, overlaid with a transparent surface representation of their tertiary structure. Proteins were chosen to exhibit a variety in size, dynamics and secondary/tertiary structure.

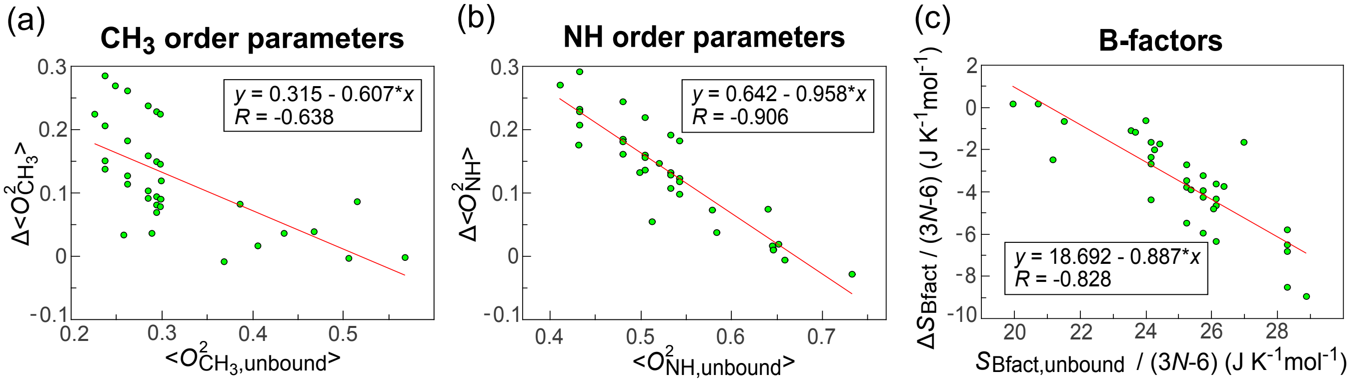

Figure 2: Change of dynamics upon complex formation. The value of different dynamics proxies before complex formation (x-axis) vs. the change upon binding (y-axis) for (a) methyl order parameters, (b) backbone-NH order parameters and (c) configurational entropy derived from crystallographic B-factors. While the order parameters in (a) and (b) are averaged over their total numbers, the B-factor entropy values are normalized by the number of internal degrees of freedom in the respective molecules.

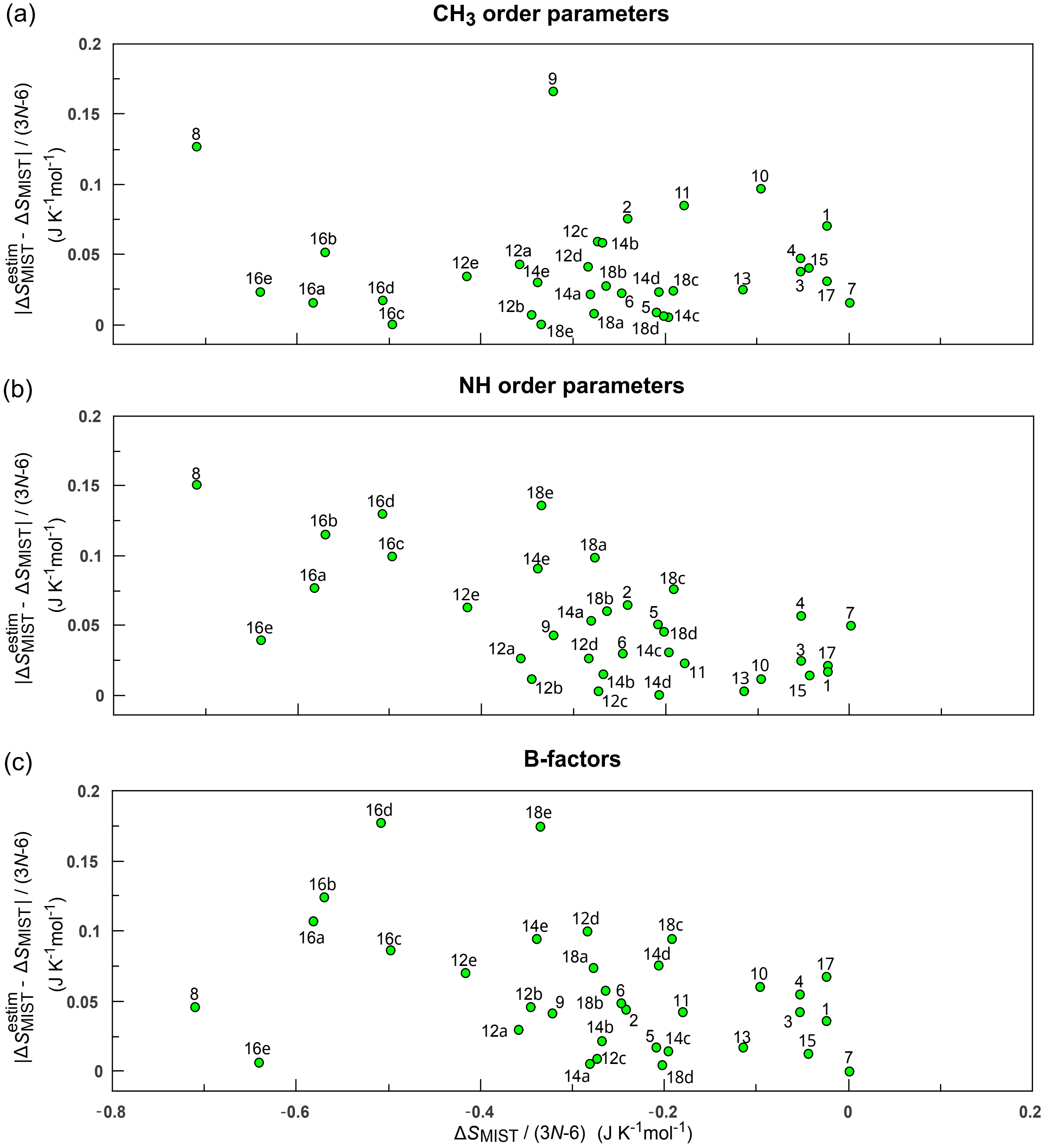

Figure 3: Absolute error introduced by estimating ΔS_{MIST} from experimental proxies as a function of the true ΔS_{MIST} normalized by the number of degrees of freedom. Absolute normalized errors $|\Delta S_{\mathrm{MIST}}^{\mathrm{estim}} - \Delta S_{\mathrm{MIST}}|/(3N-6)$ are shown, where N is the number of atoms in a given protein, which are introduced if ΔS_{MIST} is estimated from (a) methyl order parameters, (b) backbone-NH order parameters or (c) crystallographic B-factors on the basis of the linear relationships given in Figure 2 of the main text. The canonical values of $\Delta S_{\mathrm{MIST}}/(3N-6)$ are given on the x-axis and serve as a reference for the error on the y-axis.

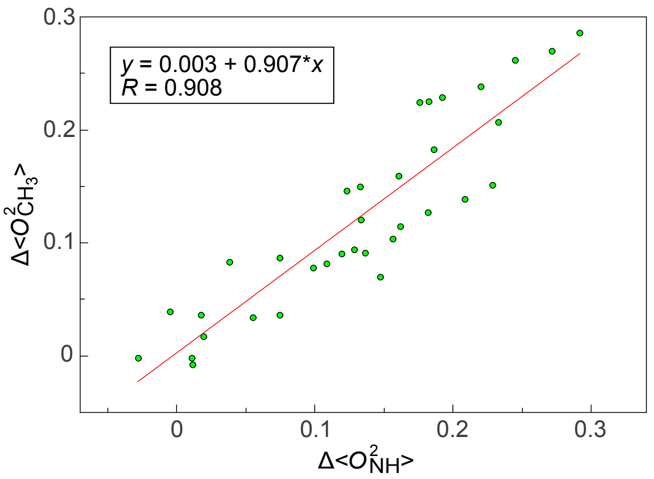

Figure 4: Order parameter comparison. $\Delta\langle O^2_{\mathrm{NH}}\rangle$ vs. $\Delta\langle O^2_{\mathrm{CH_3}}\rangle$ upon complex formation for the set of simulated proteins.

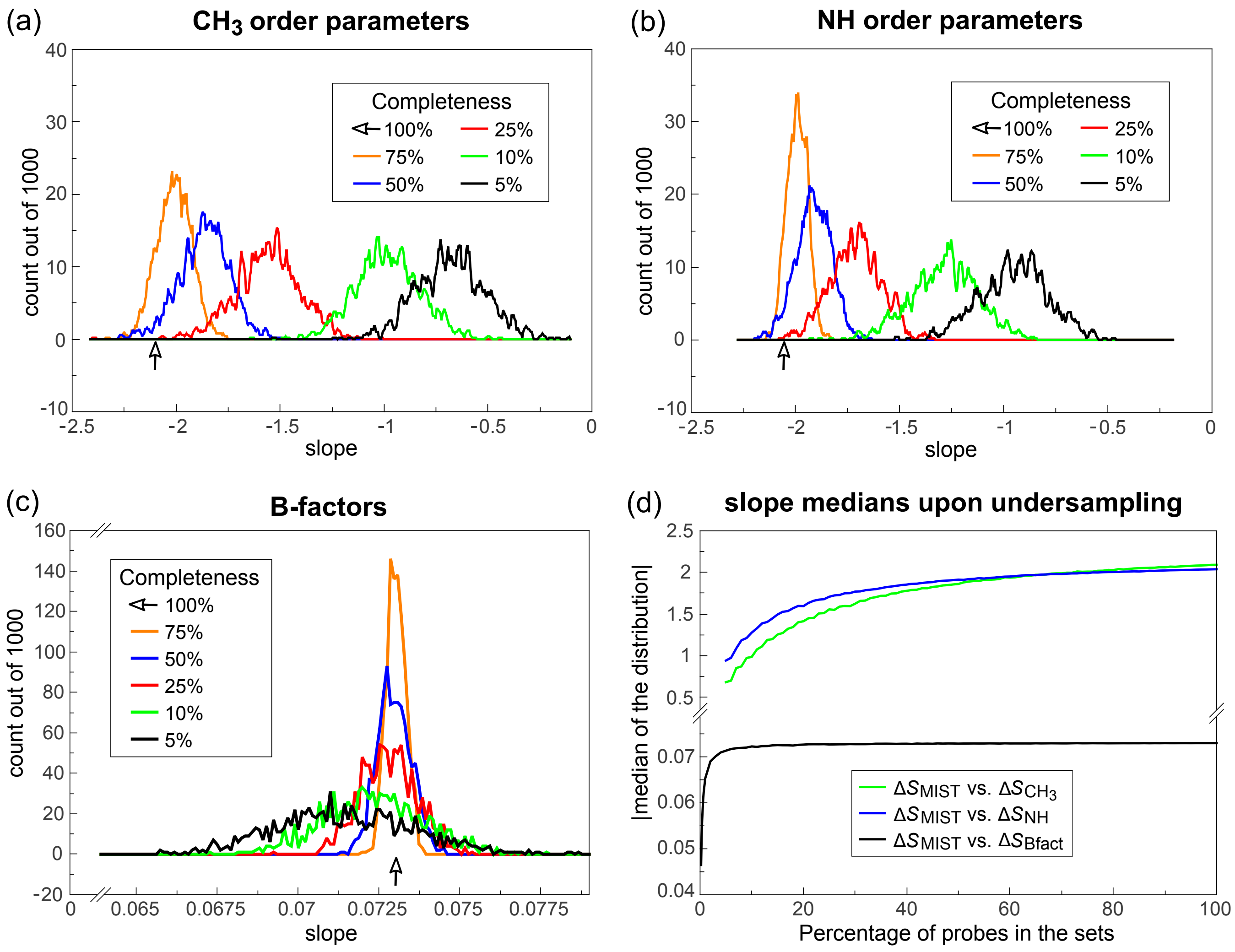

Figure 5: Dependence of the slopes between ΔS_{MIST} and different entropy proxies on the completeness of the set of experimental reporters. Distributions of slopes from linear regression between ΔS_{MIST}, evaluated for the full set of degrees of freedom, and the under-sampled (a) $\Delta\langle O^2_{\mathrm{CH_3}}\rangle$, (b) $\Delta\langle O^2_{\mathrm{NH}}\rangle$, or (c) $\Delta S_{\mathrm{Bfact}}$ over the set of 34 binding processes. The degree of undersampling is given in the inset. All values are based on the changes upon complex formation, evaluated separately for each constituent and normalized by the number of degrees of freedom for ΔS_{MIST} and $\Delta S_{\mathrm{Bfact}}$. The arrow marks the slope when taking the full set of reporters into account. (d) Absolute values of the medians of Pearson R histograms as a function of the degree of undersampling.

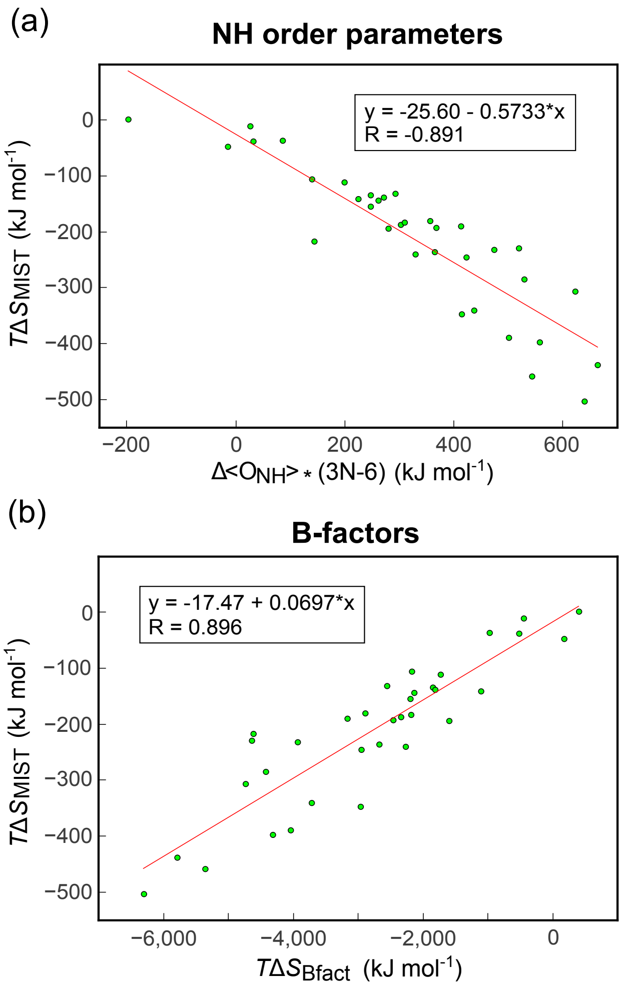

Figure 6: Comparison between experimentally accessible measures of protein dynamics and ΔS_{MIST}. (a) $\Delta\langle O^2_{\mathrm{NH}}\rangle$ vs. ΔS_{MIST}, (b) $\Delta S_{\mathrm{Bfact}}$ vs. ΔS_{MIST}. $\Delta\langle O^2_{\mathrm{CH_3}}\rangle$ vs. ΔS_{MIST} is shown in the main text as Fig. 4(a). All values reflect the entropy changes upon complex formation, evaluated separately for each individual protein. To obtain an extensive quantity, $\Delta\langle O^2_{\mathrm{NH}}\rangle$ has been scaled by the number of degrees of freedom. To arrive at the associated free-energetic contributions, entropy values have been multiplied by the temperature of the simulations, i. e. 300 K. For each comparison, we provide the least-squares linear fit and the associated Pearson correlation coefficient R.

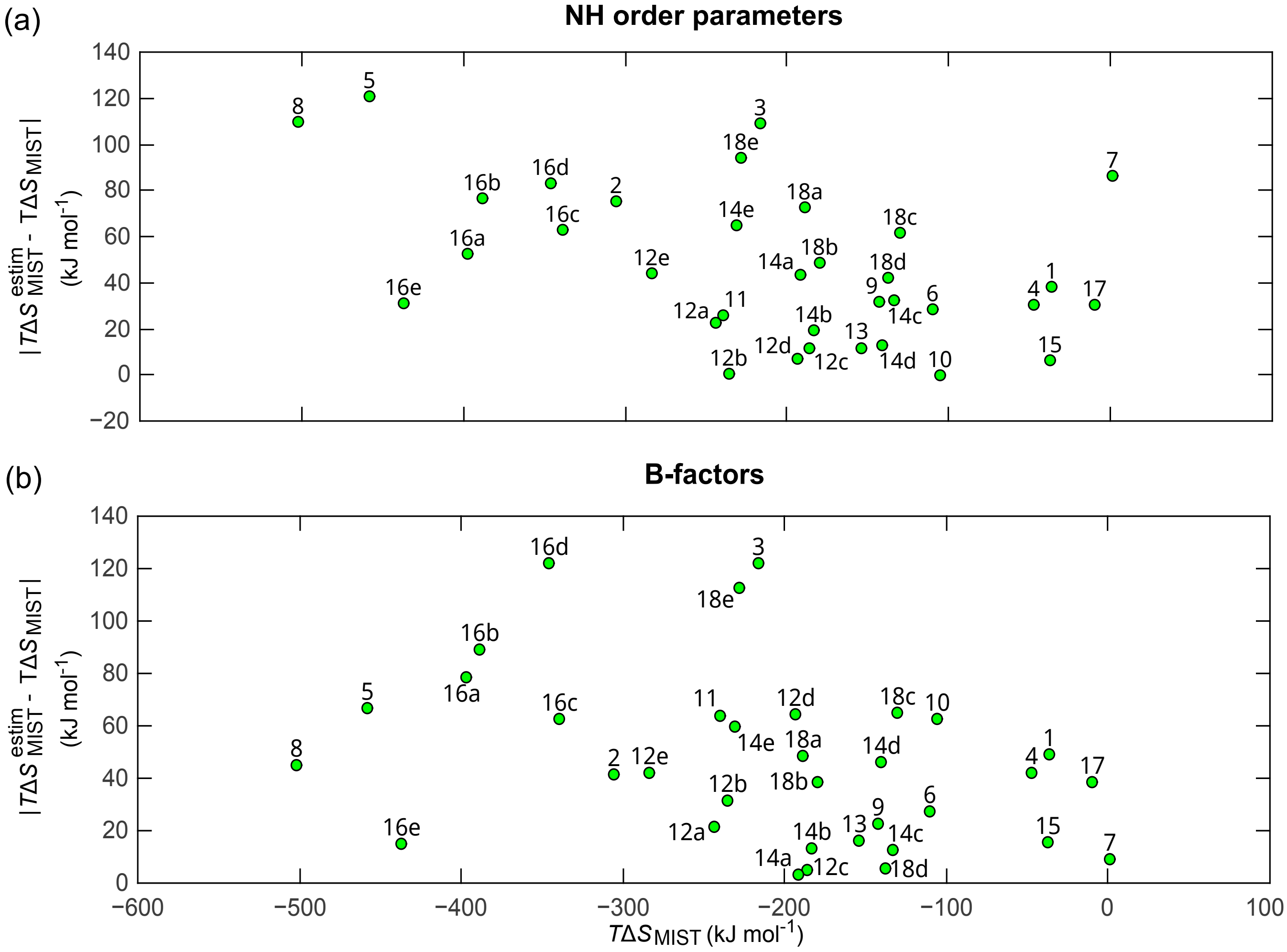

Figure 7: Absolute error introduced by estimating $T\Delta S_{\mathrm{MIST}}$ from experimental proxies as compared to the true $T\Delta S_{\mathrm{MIST}}$. Absolute errors $|T\Delta S^{\mathrm{estim}}_{\mathrm{MIST}} - T\Delta S_{\mathrm{MIST}}|$ are shown, which are introduced if $T\Delta S_{\mathrm{MIST}}$ is estimated from (a) backbone-NH order parameters or (b) crystallographic B-factors on the basis of the linear relationships given in Figure 2 of the main text. The canonical values of $T\Delta S_{\mathrm{MIST}}$ are given on the x-axis and serve as a reference for the error on the y-axis. The analogous graph for methyl order parameters is given in Fig. 4(b) of the main text.

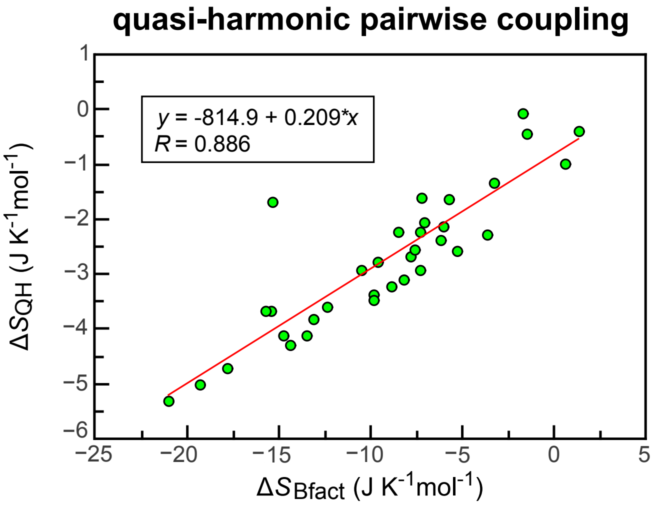

Figure 8: Effect of pairwise couplings in the QH approximation. Shown are configurational entropy changes upon binding for every protein in the simulated set, whereby coupling corrections of pairwise order are included on the y-axis and excluded on the x-axis. In the inset, we provide the least-squares linear fit and the associated Pearson correlation coefficient.

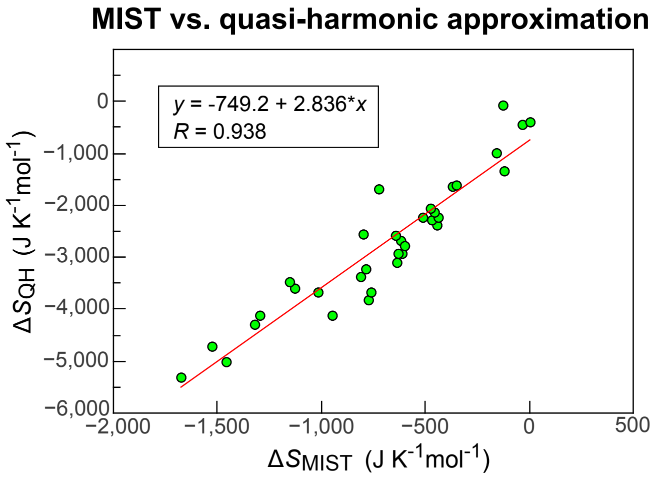

Figure 9: Comparison between the QH and the MIST approximations. The relationship between MIST values in BAT coordinates and the QH values in Cartesian coordinates is shown, both including the coupling contributions up to second order. In the inset, we provide the least-squares linear fit and the associated Pearson correlation coefficient.

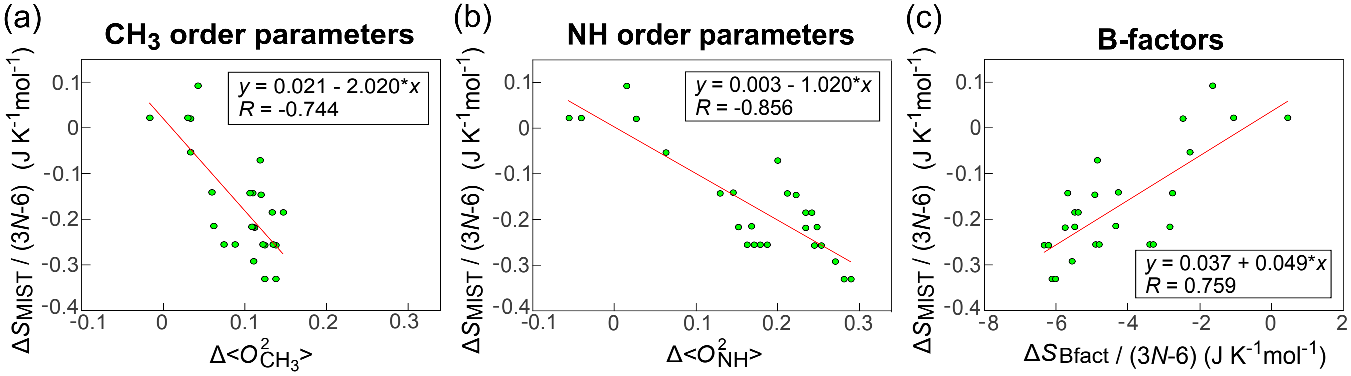

Figure 10: Comparison of ΔS_{MIST} with experimentally accessible measures of protein dynamics for UBM2 binding to UBQ. Five different trajectories for unbound UBM2 as well as five different trajectories for the UBQ-UBM2 complex yield 25 data points. (a) $\Delta\langle O^2_{\mathrm{CH_3}}\rangle$ vs. ΔS_{MIST}, (b) $\Delta\langle O^2_{\mathrm{NH}}\rangle$ vs. ΔS_{MIST} and (c) $\Delta S_{\mathrm{Bfact}}$ vs. ΔS_{MIST}. All values reflect the entropy changes for UBM2 upon complex formation, evaluated separately for each individual copy of the protein. The $\Delta S_{\mathrm{Bfact}}$ and ΔS_{MIST} values are normalized by the number of degrees of freedom in the protein ($3N-6$, where N is the number of atoms). For each comparison, we provide the least-squares linear fit and the associated Pearson correlation coefficient R.

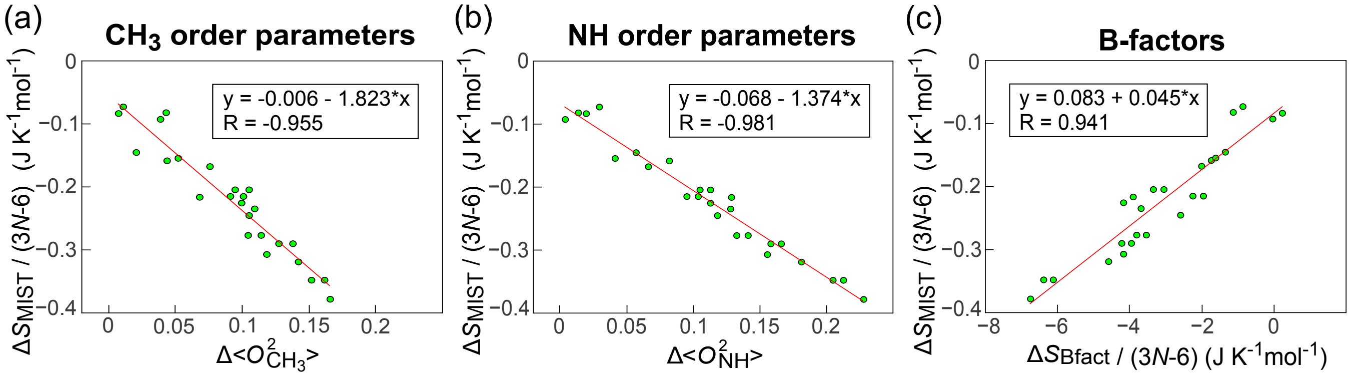

Figure 11: Comparison of ΔS_{MIST} with experimentally accessible measures of protein dynamics for UBQ binding to UBM2. Five different trajectories for unbound UBQ as well as five different trajectories for the UBQ-UBM2 complex yield 25 data points. (a) $\Delta\langle O^2_{\mathrm{CH_3}}\rangle$ vs. ΔS_{MIST}, (b) $\Delta\langle O^2_{\mathrm{NH}}\rangle$ vs. ΔS_{MIST} and (c) S_{Bfact} vs. ΔS_{MIST} . All values reflect the entropy changes for UBQ upon complex formation, evaluated separately for each individual copy of the protein. The $\Delta S_{\mathrm{Bfact}}$ and ΔS_{MIST} values are normalized by the number of degrees of freedom in the protein ($3N-6$, where N is the number of atoms). For each comparison, we provide the least-squares linear fit and the associated Pearson correlation coefficient R.

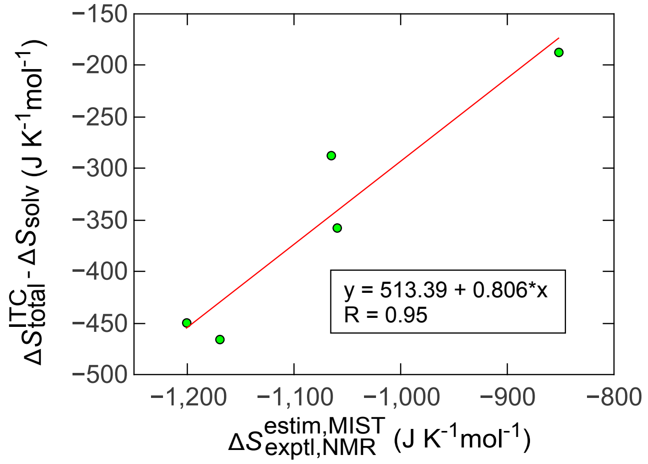

Figure 12: Comparison of experimental configurational entropy changes with estimates from the MIST approximation for calmodulin binding to different ligands. On the x-axis, the numerically determined slope from Fig. 2 (a) in the main text was used to estimate configurational entropy changes according to the MIST approximation, but by using experimental methyl order parameters of calmodulin from Caro et al.[1] To match numerical order parameters, the experimental order parameters where scaled by a factor of 0.64.[2] The y-axis shows the respective total entropy changes minus the solvent entropy changes, as determined experimentally in Caro et al.[1]

References

(1) Caro, J. A.; Harpole, K. W.; Kasinath, V.; Lim, J.; Granja, J.; Valentine, K. G.; Sharp, K. A.; Wand, A. J. *PNAS* **2017**, *114*, 6563–6568.

(2) Kasinath, V.; Sharp, K. A.; Wand, A. J. *J. Am. Chem. Soc.* **2013**, *135*, 15092–15100.
